# SUrvey of Guideline Adherence for Treatment of Systolic Heart Failure in Real World (SUGAR): A Multi-Center, Retrospective, Observational Study

**DOI:** 10.1371/journal.pone.0086596

**Published:** 2014-01-27

**Authors:** Byung-Su Yoo, Jaewon Oh, Bum-Kee Hong, Dae-Hee Shin, Jang-Ho Bae, Dong Heon Yang, Wan-Joo Shim, Hyung-seop Kim, Su-Hong Kim, Jin-Oh Choi, Woo-Jung Chun, Choong-Won Go, Hyun-Jae Kang, Sang Hong Baek, Jang-hyun Cho, Suk-Keun Hong, Joon-Han Shin, Seok-Kyu Oh, Wook-Bum Pyun, Jun Kwan, Young-Joon Hong, Jin-Ok Jeong, Seok-Min Kang, Dong-Ju Choi, on behalf of the SUGAR Study

**Affiliations:** 1 Division of Cardiology, Yonsei University Wonju Severance Christian Hospital, Wonju, Korea; 2 Division of Cardiology, Yonsei University Severance Hospital, Seoul, Korea; 3 Division of Cardiology, Gangnam Severance Hospital, Seoul, Korea; 4 Division of Cardiology, Gangneung Asan Hospital, Gangneung, Korea; 5 Division of Cardiology, Konyang University Hospital, Daejeon, Korea; 6 Division of Cardiology, Kyungpook National University Hospital, Daegu, Korea; 7 Division of Cardiology, Korea University Anam Hospital, Seoul, Korea; 8 Division of Cardiology, Keimyung University Dongsan Hospital, Daegu, Korea; 9 Division of Cardiology, Busan Veterans Hospital, Busan, Korea; 10 Division of Cardiology, Samsung Medical Center, Seoul, Korea; 11 Division of Cardiology, Sungkyunkwan University Samsung Changwon Hospital, Changwon, Korea; 12 Division of Cardiology, Inje University Sanggye Paik Hospital, Seoul, Korea; 13 Division of Cardiology, Seoul National University Hospital, Seoul, Korea; 14 Division of Cardiology, Seoul St. Mary's Hospital of the Catholic University of Korea, Seoul, Korea; 15 Division of Cardiology, St. Carollo Hospital, Suncheon, Korea; 16 Division of Cardiology, Sejong General Hospital, Bucheon, Korea; 17 Division of Cardiology, Ajou University Hospital, Suwon, Korea; 18 Division of Cardiology, Wongwang University Hospital, Iksan, Korea; 19 Division of Cardiology, Ewha Womans University Mokdong Hospital, Seoul, Korea; 20 Division of Cardiology, Inha University Hospital, Incheon, Korea; 21 Division of Cardiology, Chonnam National University Hospital, Gwangju, Korea; 22 Division of Cardiology, Chungnam National University Hospital, Daejeon, Korea; 23 Division of Cardiology, Seoul National University Bundang Hospital, Seongnam, Korea; Universidad Peruana Cayetano Heredia, Peru

## Abstract

**Background:**

Clinical practice guidelines have been slowly and inconsistently applied in clinical practice, and certain evidence-based, guideline-driven therapies for heart failure (HF) have been significantly underused. The purpose of this study was to survey guideline compliance and its effect on clinical outcomes in the treatment of systolic HF in Korea.

**Method and Results:**

The SUrvey of Guideline Adherence for Treatment of Systolic Heart Failure in Real World (SUGAR) trial was a multi-center, retrospective, observational study on subjects with systolic HF (ejection fraction <45%) admitted to 23 university hospitals. The guideline adherence indicator (GAI) was defined as a performance measure on the basis of 3 pharmacological classes: angiotensin-converting enzyme inhibitor (ACEI) or angiotensin receptor II blocker (ARB), beta-blocker (BB), and aldosterone antagonist (AA). Based on the overall adherence percentage, subjects were divided into 2 groups: those with good guideline adherence (GAI ≥50%) and poor guideline adherence (GAI <50%). We included 1319 regional participants as representatives of the standard population from the Korean national census in 2008. Adherence to drugs at discharge was as follows: ACEI or ARB, 89.7%; BB, 69.2%; and AA, 65.9%. Overall, 82.7% of the patients had good guideline adherence. Overall mortality and re-hospitalization rates at 1 year were 6.2% and 37.4%, respectively. Survival analysis by log-rank test showed a significant difference in event-free survival rate of mortality (94.7% vs. 89.8%, p = 0.003) and re-hospitalization (62.3% vs. 56.4%, p = 0.041) between the good and poor guideline-adherence groups.

**Conclusions:**

Among patients with systolic HF in Korea, adherence to pharmacologic treatment guidelines as determined by performance measures, including prescription of ACEI/ARB and BB at discharge, was associated with improved clinical outcomes.

## Introduction

Despite extensive evidence and recommendations from clinical trials, heart failure (HF) remains a substantial cause of morbidity and mortality. Pharmacological therapy including administration of angiotensin-converting enzyme inhibitor (ACEI) or angiotensin receptor II blocker (ARB), beta-blocker (BB), and aldosterone receptor antagonist (AA) can reduce morbidity and mortality in patients with HF. However, this evidence-based, guideline-driven medication is significantly underused. Treatment guideline adherence is an important predictor of clinical deterioration.[1], [2] To enhance treatment adherence in HF patients, performance improvement programs have been established, and improvement of performance measures is important to improve patient care and outcomes.[3]–[5] However, treatment adherence varies depending on age, sex, race, and socioeconomic status.[6], [7] In addition, ethnic and racial differences in etiology, outcome, and response to therapy in HF patients have been demonstrated, so the clinical benefits of evidence-based drug therapy for different circumstances must be validated.[8]–[10] The major clinical effects of evidence-based drug therapy were evaluated especially in the western population.[1], [11]–[14] The Asia-Pacific region is very diverse in terms of living standards, ethnicity, and population. However, little is known about the adherence to HF-recommended medication in the follow-up period and its effects on clinical outcomes in patients with HF, especially in Asian countries.[15]–[17] Hence, we evaluated guideline adherence in the treatment of systolic HF and the effect of adherence to pharmacologic treatment on mortality and hospitalization rates in Korea.

## Materials and Methods

### Ethics statement

This study was approved by the institutional review boards (IRB) of Yonsei University Wonju Severance Christian Hospital, Yonsei University Severance Hospital, Yonsei University Gangnam Severance Hospital, Konyang University Hospital, Kyungpook National University Hospital, Korea University Anam Hospital, Keimyung University Dongsan Hospital, Samsung Medical Center, Samsung Changwon Hospital, Inje University Sanggye Paik Hospital, Seoul National University Hospital, Seoul St. Mary's Hospital of the Catholic University of Korea, Sejong General Hospital, Ajou University Hospital, Wongwang University Hospital, Ewha Womans University Mokdong Hospital, Inha University Hospital, Chonnam National University Hospital, Chungnam National University Hospital, and Seoul National University Bundang Hospital; and the ethical committees of Gangneung Asan Hospital, Busan Veterans Hospital, and St. Carollo Hospital. Informed consent was waived by the IRB of the 23 participating centers considering the retrospective study design, and the study protocol conforms to the ethical guidelines of the 1975 Declaration of Helsinki, as reflected in a priori approval by the institution's human research committee. Data were collected and managed by the Control of Data Committee of the study.

### Study design and study population

The SUrvey of Guideline Adherence for Treatment of Systolic Heart Failure in Real World (SUGAR) trial is a multi-center, retrospective, observational study on subjects with systolic HF (ejection fraction <45%) who were admitted to 23 university hospitals in Korea (clinical trial registration: URL: http://www.clinicaltrials.gov., Unique identifier: NCT01390935). The total target was 1319 patients, of which regional participants were recruited as a representative population of the standard population distribution reported in the Korean national census in 2008. Inclusion criteria were (i) age ≥20, (ii) admission to hospital with systolic HF (left ventricular ejection fraction [LVEF] <45%) in 2009, and (iii) consecutive subjects admitted to the hospital with dyspnea and verification of HF by clinical findings from January 2009 to December 2009. We excluded patients who died during hospitalization and patients with inadequate echocardiographic and clinical data. Data on demographic features, medical history, clinical characteristics, initial evaluations, therapeutic management, clinical follow up, vital status, re-admission to hospital, and major cardiovascular events was collected. Chronic renal insufficiency was defined as serum creatinine level ≥2.0 mg/dL or use of renal replacement therapy. Researchers from each hospital who collected study data referred to homogeneous case-report forms.

### Definition of treatment adherence

Performance measures were modified using the definitions defined by the American College of Cardiology/American Heart Association clinical performance measures for adults with chronic HF, the Joint Commission on Accreditation of Healthcare Organizations, the Organized Program to Initiate Lifesaving Treatment in Hospitalized Patients with Heart Failure (OPTIMIZE-HF) study, and the Medical Management of Chronic Heart Failure in Europe and Its Related Costs (MAHLER) survey.[1]–[3] We added a performance measure for AAs, as suggested by recent European Society of Cardiology (ESC) HF guidelines, because they have been proven to benefit patients with HF.[5], [18] The guideline adherence indicator (GAI) such as 0/3, 1/3, 2/3, and 3/3 was defined as a modified validated performance measure on the basis of 3 pharmacological classes (ACEI or ARB, BB, and AA).[1], [11], [12] We divided patients into 2 groups: those with good guideline adherence (GAI ≥50%, 2/3, and 3/3) or poor guideline adherence (GAI <50%, 0/3, and 1/3), based on overall GAI.

### Clinical outcomes

Ninety-day and 1-year mortality, re-hospitalization, and combined mortality/re-hospitalization rates were collected. Mortality was defined as death from any cause. Re-hospitalization was defined as an admission by aggravated HF after survival discharge.

### Statistical analyses

Statistical analyses were performed using SPSS version 21.0 (IBM, USA). Continuous variables were expressed as the mean ± standard deviation. Categorical variables were expressed as absolute numbers and percentages. To adjust for significant covariates, multivariable models were developed for post-discharge all-cause mortality and morbidity. We used binary logistic regression models to estimate unadjusted and adjusted relationships between each performance measure and patient outcome. The unadjusted models included only each component of the performance measures as a predictor. The adjusted models were controlled for baseline demographic characteristics and clinical characteristics. These included age; sex; history of HF, ischemic heart failure, diabetes mellitus, and peripheral vascular disease; diastolic blood pressure; blood urea nitrogen (BUN); serum creatinine, sodium, and potassium levels; hemoglobin levels; and the New York Heart Association (NYHA) functional classification. Kaplan-Meier analyses were used to compare between endpoints such as mortality, re-hospitalization, and mortality/re-hospitalization, and each component of performance measures/guideline adherence. The log-rank test was used to test for differences in unadjusted survival curves. A two-sided P value of <0.05 was considered significant. Forest plots were performed using GraphPad Prism version 5.03 (GraphPad Software, La Jolla, CA, USA).

## Results

### Baseline characteristics of patients

**Figure 1:**
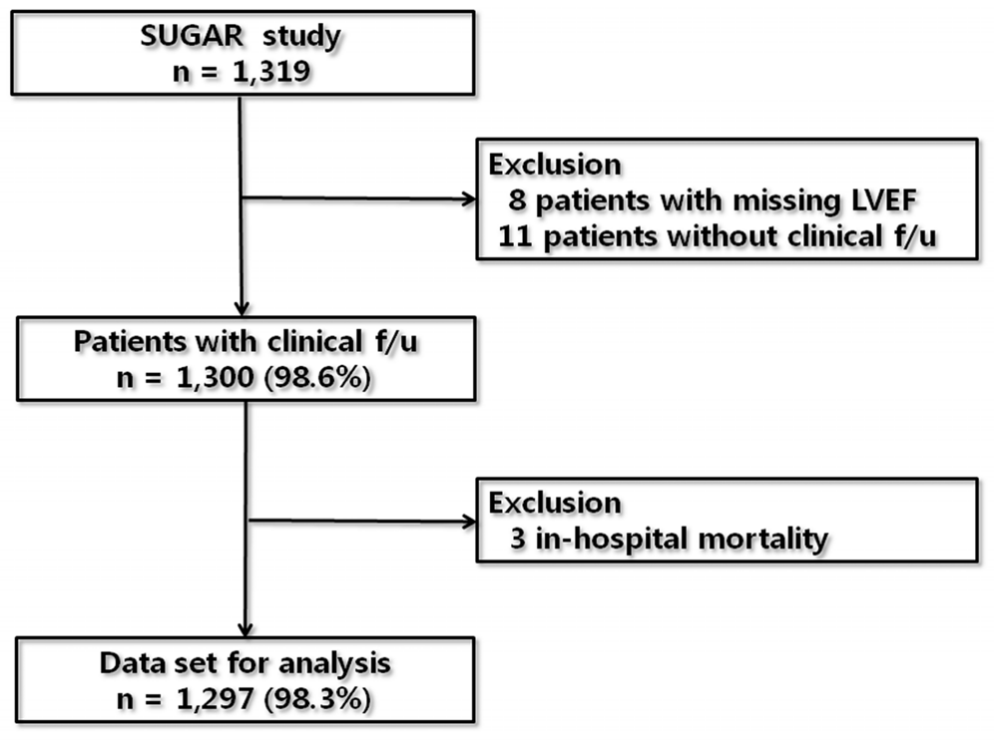
Dataset for the study population. LVEF, left ventricular ejection fraction; f/u, follow-up.

The SUGAR study enrolled 1319 patients. Among this population, 8 patients with inadequate echocardiographic data for LVEF and 11 patients without clinical follow-up data were excluded. Among the 1300 patients with clinical follow up, 3 in-hospital mortality cases were excluded to restrict the influence of underlying disease severity on the benefits of discharge medications. Finally, 1297 patients (98.3%) who survived during hospitalization and had follow-up data were evaluated (Figure 1).

The baseline clinical and laboratory characteristics of the study population are presented in Table 1. The median age of the study population was 69 years, and the percentage of men was 56.3%. The percentage of patients graded as NYHA classification III or IV was 68.0%, and 40.2% of the patients had ischemic heart disease. The median ejection fraction on echocardiography was 29.7%. At hospital admission, 29.5% of the patients had atrial fibrillation. The most common comorbid condition was hypertension (58.4%), and 43.3% of the patients had a history of HF.

**Table 1:** Baseline characteristics of the study population.

Variables	Patients (n = 1297)
**Men, n (%)**	730 (56.3)
**Age, median (IQR), years**	69 (58 – 78)
**Anthropometric data, mean ± SD**	
Height, cm	160.4±10.0
Weight, kg	60.0±12.9
BMI, kg/m^2^	23.3±3.9
**Vital signs at admission, mean ± SD**	
SBP, mmHg	129.5±28.5
DBP, mmHg	78.9±17.4
HR, beats per minute	92.3±24.2
**NYHA classification, n (%)**	
Class II	415 (32.0)
Class III	608 (46.9)
Class IV	274 (21.1)
**Ischemic heart failure, n (%)**	522 (40.2)
CABG	59 (4.5)
PCI or PTCA	227 (17.5)
**Comorbid conditions, n (%)**	
Hypertension	758 (58.4)
Diabetes mellitus	454 (35.0)
Dyslipidemia	324 (25.0)
Previous heart failure	562 (43.3)
Myocardial infarction	235 (18.1)
PAOD	32 (2.5)
Chronic pulmonary disease	81 (6.2)
Chronic renal insufficiency	158 (12.2)
Cerebral infarction	139 (10.7)
Smokers/Past-smokers	293 (22.6)/302 (23.3)
CABG	59 (4.5)
PCI	227 (17.5)
Valve surgery	34 (2.6)
**Laboratory findings at admission (mean ± SD)**	
Hemoglobin, g/dL	12.6±2.3
BUN, mg/dL	25.9±16.3
Creatinine, mg/dL	1.46±1.14
Glucose, mg/dL	157.4±90.1
Total cholesterol, mg/dL	162.2±66.7
Na, mmol/L	138.6±4.9
K, mmol/L	4.4±0.7
CK-MB, ng/mL	10.8±38.9
Troponin I, ng/mL (n = 805)	1.26±6.43
BNP, median (IQR), pg/mL (n = 472)	1083 (607 – 1850)
NT-proBNP, median (IQR), pg/mL (n = 626)	6613 (3458 – 14467)
**ECG finding at admission, n (%)**	
Atrial fibrillation	383 (29.5)
LBBB	148 (11.4)
**Echocardiographic finding (mean ± SD)**	
LVEF, %	29.7±8.8
LVEDD, mm	59.1±13.8
LA AP diameter, mm	45.0±15.4
**Discharge medications, n (%)**	
ACEI/ARB	1163 (89.7)
BB	897 (69.2)
AA	855 (65.9)

AA, aldosterone receptor antagonist; ACEI, angiotensin-converting enzyme inhibitor; ARB, angiotensin II receptor blocker; BB, beta-blocker; BMI, body mass index; BNP, b-type natriuretic peptide; BUN, blood urea nitrogen; CABG, coronary bypass graft surgery; CK-MB, creatine kinase-MB fraction; DBP, diastolic blood pressure; ECG, electrocardiography; HR, heart rate; IQR, interquartile range; LA AP, left atrium anterior-posterior; LBBB, left bundle branch block; LVEDD, left ventricular end diastolic dimension; LVEF, left ventricular ejection fraction; NT-proBNP, N-terminal-proBNP; NYHA, New York Heart Association; PAOD, peripheral artery obstructive disease; PCI, percutaneous coronary intervention; PTCA, percutaneous transluminal coronary angioplasty; SBP, systolic blood pressure; SD, standard deviation

### Performance measures and clinical outcomes

At discharge, treatment adherence was highest with ACEI or ARB (89.7%), followed by BB (69.2%) and AA (65.9%). The most commonly prescribed ACEI was ramipril (22.5%), followed by perindopril (12.5%); the most commonly prescribed ARB was candesartan (14.0%), followed by losartan (11.7%); and the most commonly prescribed BB was carvedilol (74.1%), followed by bisoprolol (17.7%) (Table 2). The only AA administrated was spironolactone, because eplerenone was not available in Korea until now. The following GAI values were calculated: 1.5% (0/3, n = 20), 15.8% (1/3, n = 205), 39.0% (2/3, n = 506), and 43.6% (3/3, n = 566). Finally, 82.7% of the patients had good guideline adherence (n = 1072).

**Table 2:** Drug adherence.

Measure	Adherence, n (%)
**ACEI or ABR at discharge (n = 1380)**	
Ramipril	310 (22.5)
Candesartan	193 (14.0)
Losartan	162 (11.7)
Perindopril	173 (12.5)
Captopril	147 (10.7)
Valsartan	130 (9.4)
Other	265 (19.2)
**β-Blocker at discharge (n = 897)**	
Carvedilol	665 (74.1)
Bisoprolol	159 (17.7)
Atenolol	39 (4.3)
Other	34 (3.8)
**Aldosterone antagonist at discharge (n = 855)**	
Spironolactone	855 (100)

ACEI, angiotensin-converting enzyme inhibitor; ARB, angiotensin receptor II blocker

After exclusion of in-hospital mortality cases, survivors (n = 1297) at discharge were followed up for a median of 272 days (interquartile range, 65–366 days). Among these survivors, the mortality rate was 2.3% (n = 30) at 90 days and 6.2% (n = 80) at 1 year. The prevalence of re-hospitalization was 18.8% (n = 244) at 90 days and 37.4% (n = 485) at 1 year. The prevalence of combined mortality/re-hospitalization was 19.6% (n = 254) at 90 days and 38.7% (n = 502) at 1 year. Table 3 shows the relationships between treatment performance measures and clinical outcomes at 90 days and 1 year. Before adjustment, ACEI or ARB use at discharge was significantly associated with re-hospitalization and combined mortality/re-hospitalization rates at 90 days and 1 year. However, BB use at discharge was only significantly associated with mortality rates at 90 days and 1 year. Moreover, AA use at discharge was not significantly associated with any clinical outcomes. After adjusting for baseline demographic an clinical characteristics, BB administration was significantly associated with mortality (hazard ratio [HR], 0.371; 95% confidence interval [CI], 0.175–0.786; p = 0.010) at 90 days, and AA administration was significantly associated with increased re-hospitalization (HR, 1.366; 95% CI, 1.016–1.838; p = 0.039) at 90 days. When we adopted AA eligibility (estimated glomerular filtration rate >30 mL • min^−1^ • 1.73 m^−2^ and serum potassium <5.0 mmol/L) for analysis, there was no statistical significance (HR, 1.465; 95% CI, 0.918–2.338, p = 0.109).

**Table 3:** Relationship between performance measures/guideline adherence at discharge and clinical outcomes.

	Unadjusted		Adjusted*	
	HR (95% CI)	p	HR (95% CI)	p
**Mortality at 90 days, n = 30 (2.3%)**				
ACEI or ARB at discharge	0.458 (0.187–1.120)	0.087	0.609 (0.240–1.546)	0.297
BB at discharge	0.332 (0.161–0.683)	0.003	0.371 (0.175–0.786)	0.010
AA at discharge	0.579 (0.283–1.186)	0.135	0.802 (0.363–1.772)	0.586
Poor vs. good guideline adherence	0.204 (0.100–0.417)	< 0.001	0.269 (0.127–0.570)	0.001
**Re-hospitalization at 90 days, n = 244 (18.8%)**				
ACEI or ARB at discharge	0.660 (0.459–0.948)	0.025	0.797 (0.549–1.157)	0.233
BB at discharge	0.910 (0.696–1.190)	0.490	0.971 (0.734–1.286)	0.839
AA at discharge	1.116 (0.852–1.462)	0.424	1.366 (1.016–1.838)	0.039
Poor vs. good guideline adherence	0.854 (0.621–1.173)	0.330	1.025 (0.733–1.433)	0.884
**Mortality or re-hospitalization at 90 days, n = 254 (19.6%)**				
ACEI or ARB at discharge	0.668 (0.468–0.955)	0.027	0.815 (0.565–1.177)	0.275
BB at discharge	0.898 (0.691–1.167)	0.420	0.966 (0.734–1.270)	0.803
AA at discharge	1.161 (0.889–1.516)	0.272	0.815 (0.565–1.177)	0.275
Poor vs. good guideline adherence	0.852 (0.624–1.163)	0.313	1.027 (0.740–1.425)	0.876
**Mortality at 1 year, n = 80 (6.2%)**				
ACEI or ARB at discharge	0.632 (0.342–1.167)	0.143	0.799 (0.427–1.495)	0.482
BB at discharge	0.575 (0.369–0.895)	0.014	0.668 (0.422–1.057)	0.085
AA at discharge	0.989 (0.622–1.572)	0.963	1.127 (0.683–1.861)	0.639
Non-compliance vs. compliance	0.488 (0.301–0.792)	0.004	0.574 (0.347–0.951)	0.031
**Re-hospitalization at 1 year, n = 485 (37.4%)**				
ACEI or ARB at discharge	0.676 (0.518–0.881)	0.004	0.806 (0.614–1.057)	0.119
BB at discharge	0.895 (0.739–1.083)	0.253	0.972 (0.795–1.189)	0.785
AA at discharge	1.047 (0.867–1.265)	0.634	1.177 (0.960–1.444)	0.117
Poor vs. good guideline adherence	0.792 (0.633–0.991)	0.042	0.916 (0.724–1.160)	0.468
**Mortality or re-hospitalization at 1 year, n = 502 (38.7%)**				
ACEI or ARB at discharge	0.690 (0.531–0.897)	0.006	0.831 (0.635–1.087)	0.176
BB at discharge	0.890 (0.738–1.073)	0.223	0.973 (0.798–1.185)	0.782
AA at discharge	1.055 (0.876–1.270)	0.575	1.184 (0.968–1.447)	0.100
Poor vs. good guideline adherence	0.794 (0.637–0.991)	0.041	0.924 (0.733–1.165)	0.504

ACEI, angiotensin-converting enzyme inhibitor; ARB, angiotensin II receptor blocker; BB, beta-blocker; AA, aldosterone antagonist; CI, confidence interval; HR, hazard ratio; NYHA, New York Heart Association

* Adjusted for age; history of heart failure, ischemic heart failure, diabetes mellitus, and peripheral vascular disease; diastolic blood pressure; blood urea nitrogen; serum creatinine, sodium, and potassium; hemoglobin levels; and NYHA functional classification.

**Figure 2:**
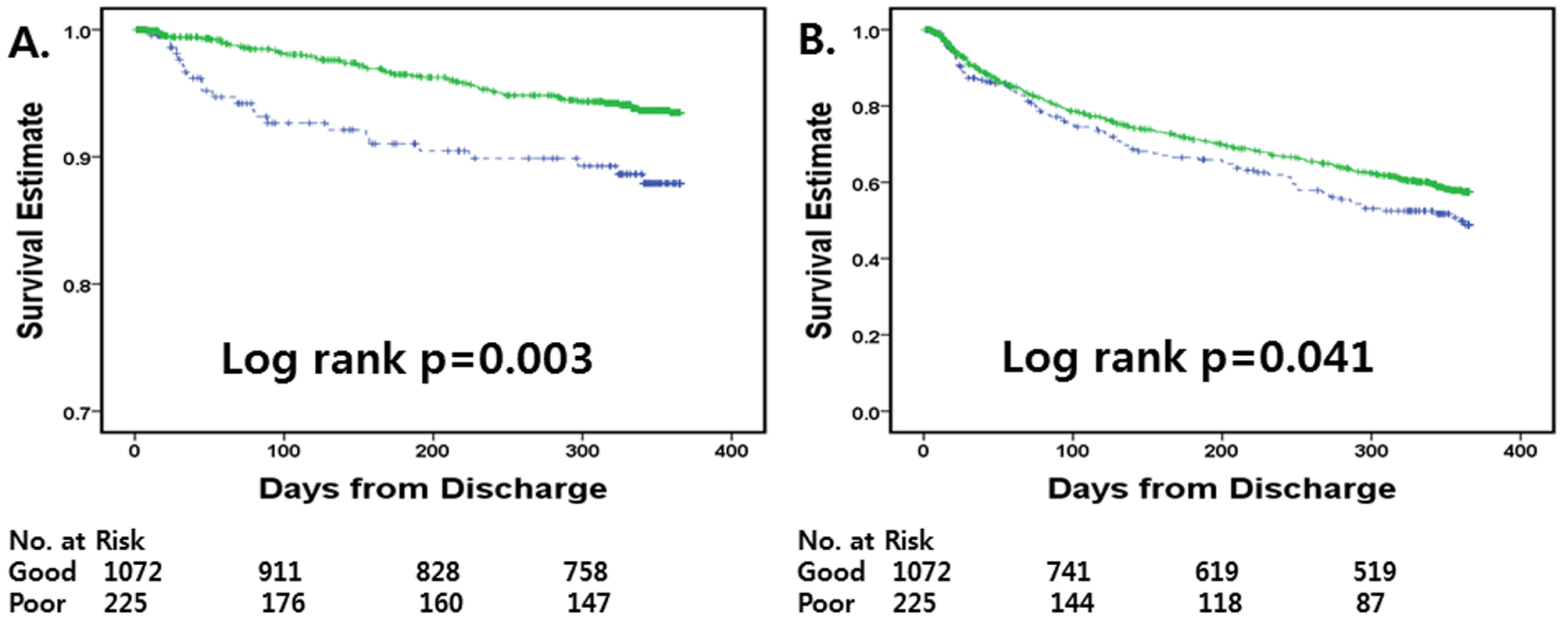
Unadjusted event-free curves for overall mortality and re-hospitalization in HF patients with good and poor guideline adherence at discharge. (A) overall mortality and (B) re-hospitalization. HF, heart failure; Good, good guideline adherence; Poor, poor guideline adherence; Line, good guideline adherence; dotted line, poor guideline adherence.

Figure 2 shows the Kaplan-Meier curves for mortality and re-hospitalization according to guideline adherence for 1 year after discharge. Compared to poor guideline adherence, good guideline adherence was associated with reduced mortality (94.7% vs. 89.8%, log-rank p = 0.003, Figure 2A) and re-hospitalization rates (62.3% vs. 56.4%, log-rank p = 0.041, Figure 2B) during the 1-year follow up. In unadjusted Cox regression analysis, good guideline adherence was related to lower mortality at 90 days and lower mortality, re-hospitalization, and combined morality/re-hospitalization at 1 year. After adjustment, good guideline adherence was associated with reduced mortality at 90 days (HR, 0.269; 95% CI, 0.127–0.570, p = 0.001) and 1 year (HR, 0.574; 95% CI, 0.347–0.951, p = 0.031) (Table 3).

**Figure 3:**
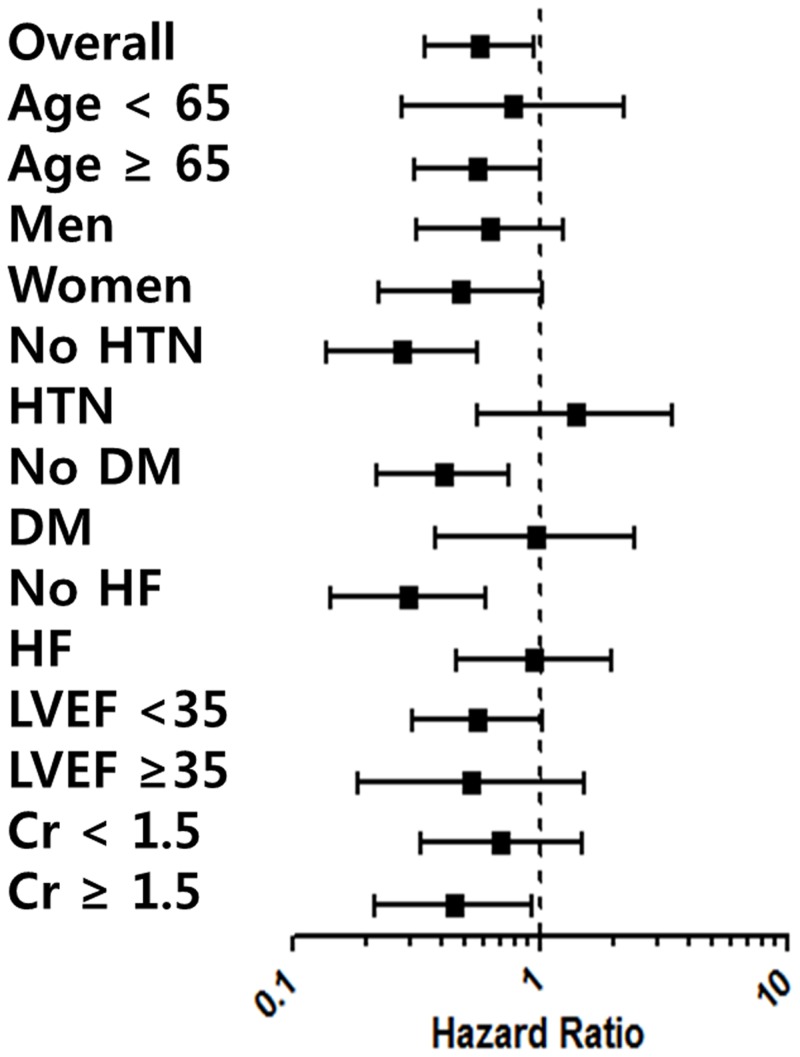
Adjusted Cox-regression analysis for 1-year mortality at in subgroups according to guideline adherence: Forest plot. HTN, hypertension; DM, diabetes mellitus; HF, heart failure; LVEF, left ventricular ejection fraction; Cr, creatinine.

Figure 3 shows forest plots for adjusted Cox regression analysis for mortality at 1 year in subgroups according to guideline adherence. Good guideline adherence was related to lower mortality at 1 year, especially in the patients without hypertension (HR, 0.279; 95% CI, 0.137–0.565, p < 0.001), without diabetes mellitus (HR, 0.408; 95% CI, 0.220–0.757, p < 0.001), and without prior HF (HR, 0.295; 95% CI, 0.144–0.606, p < 0.001).

## Discussion

This is the first national representative data in systolic HF patients in Asia. Moreover, we evaluated the overall adherence to HF performance measures as well as the relationship between guideline adherence and clinical outcomes in hospitalized systolic HF patients in Korea for the first time. We found that the guideline adherence, according to performance measures, in “real practice” was relatively high compared to that of a previous retrospective registry, Korean Heart Failure Registry (KorHF Registry) in Korea.[17] We also found that prescription of ACEI or ARB and BB at discharge was associated with reduced early and mid-term clinical outcomes, but the use of AA was associated with increased early re-hospitalization rate. These findings are consistent with recent data from Western studies and provide important implications for improvement in quality of life.[19]

Most large-scale registry databases mainly have information on patients in the USA and Europe.[20]–[23] Recently, some limited information has become available on the characteristics and outcomes of hospitalized HF patients in Asia.[15], [17], [24], [25] The baseline characteristics and administration rates of ACEI or ARB and BB in SUGAR are comparable to those of other registries, such as the Acute Decompensated Heart Failure National Registry,[21] OPTIMIZE-HF,[23] EuroHeart Failure Survey,[20], [22] or the Japanese Cardiac Registry of Heart Failure in Cardiology.[15] The administration rates of AAI/ARB and BB were remarkably increased in our study compared with the results of the KorHF Registry data (ACEI/ARB, 68.0% to 89.7%; BB, 40.9% to 69.2%).[17], [24] In particular, compared with other HF registry studies, the use of AA was higher in SUGAR, consistent with previous Korean registry findings.[17], [24]

There has been increasing evidence of a relationship between evidence-based performance measures and outcomes, especially in Western countries.[1], [11]–[14], [26] However, further efforts are needed to clarify this relationship, because of the heterogeneity of study designs, study population, and the definition of performance measures. Additionally, ethnic or racial differences must be a concern because ethnic and racial differences in etiology, outcome, and response to therapy in HF patients have been demonstrated. Our study is the first study to demonstrate the relationship between evidence-based treatment adherence and clinical outcomes in Asian countries. We found that guideline-recommended drug adherence was related with reduced short/mid-term mortality in Korean systolic HF patients. Although we adopted the current ESC guideline, which widened AA indications in HF patients with mild symptoms, we also showed the prognostic importance of guideline adherence in the fully adjusted Cox regression model, which was consistent with previous reports.[1], [11]–[14] In subgroup analysis, guideline adherence was more closely related to lower mortality in low-risk HF patients (e.g., no comorbidity, no prior HF admission history). Therefore, early, guideline-recommended medical therapy should be considered in newly diagnosed HF patients.

The Randomized Aldactone Evaluation Study (RALES) demonstrated that spironolactone reduces the risk of mortality and morbidity in patients with systolic HF.[27] In the Eplerenone in Mild Patients Hospitalization and Survival Study in Heart Failure (EMPHASIS-HF) study, eplerenone also reduced both the risk of death and hospitalization in systolic HF patients with mild symptoms.[18] Following this line of evidence, current ESC guidelines recommend the use of mineralocorticoid/AA (eplerenone and spironolactone) in HF patients with reduced LVEF.[5] However, the patients in the RALES and EMPHASIS-HF trial were clearly different from those in the current study, and the relationship between AA and clinical outcome could be different. First, AA use at discharge was associated with increased re-hospitalization at 90 days in multivariable Cox regression analysis. This may have resulted from hyperkalemia-associated morbidity, considering the relatively high administration rate of AA in our study.[28] The significant association between AA administration and increased re-hospitalization at 90 days disappeared after considering AA eligibility; thus, our study reasserted the guideline-recommended adherence to AA. Adequate administration of AA may be related to reduced hyperkalemia-associated re-hospitalization at an early period after hospitalization. Second, we could not find any clinical benefit of AA in systolic HF patients. This finding may be attributed to the lack of differences in clinical benefit between the 2/3 and 3/3 GAI groups in SUGAR (data not shown). Recent studies have also suggested that AA administration does not benefit clinical outcomes. Lund et al. reported that spironolactone was not associated with reduced mortality in Sweden, using a propensity-scored matching cohort study,[19] and Hernandez et al. also showed that AA use at hospital discharge was not associated with improved morality and increase in the risk of readmission within 30 days in older HF patients.[29] The mechanisms underlying these unanticipated findings may be related to a disparity in some pharmacologic properties of spironolactone and eplerenone (e.g., their effect on cortisol and hemoglobin A1c).[30], [31] Regarding the relatively higher use of AA in our study, there may be some discrepancy between randomized controlled trials and cohort registries. It is still not clear if this difference is true or due to a survey bias among large-scale registries. Therefore, a precise, coordinated, prospective study using an identical protocol and the same clinical criteria worldwide (including Western and Oriental populations) is required.

There were several limitations in the present study. First, this was a hospital-based, retrospective, observational study although the findings are representative of national care patterns and outcomes according to the standard population distribution reported in the Korean national census. Our study was limited by the nature of its design. Second, we performed multivariable regression analysis, but other unmeasured and hidden confounding variables such as socioeconomic factors and health-care-system factors may have influenced patient outcomes in the post-discharge period. In Korea, patients usually pay an average of 10–30% of the total medical costs that are covered by public insurance.[32] Therefore, these factors may also be important. Third, the outcomes assessed in this study were restricted to hospital re-hospitalizations and mortality. Other important outcomes, such as health-related quality of life, functional capacity, and the reason for HF patients not receiving the recommended drugs should also be considered as supplemental metrics. Finally, we did not assess non-pharmacological management. Fluid therapy during hospitalization, sodium intake, sleep apnea, and depression should be considered because these factors are associated with the prognosis. Because of the aforementioned limitations, the associations between the pharmacological treatment adherence and outcomes and its causality should be interpreted with caution.

## Conclusion

This study presents the first national representative data on performance measures in systolic HF patients in Asia. Adherence to pharmacologic treatment guidelines as determined by performance measures, including prescription of ACEI/ARB and BB at discharge, was associated with improved outcomes in patients with systolic HF in Korea.
